# Comparing Benzodithiophene Unit with Alkylthionaphthyl and Alkylthiobiphenyl Side-Chains in Constructing High-Performance Nonfullerene Solar Cells

**DOI:** 10.3390/polym12081673

**Published:** 2020-07-27

**Authors:** Ruyi Xie, Li Song, Zhihui Zhao

**Affiliations:** 1 School of Textiles and Clothing, Qingdao University, 308 Ningxia Road, Qingdao 266071, China; 15844215476@163.com; 2 Key Laboratory Clean Dyeing and Finishing Technology Zhejiang, Shaoxing University, Shaoxing 312000, China; 3 Collaborative Innovation Center for Eco-Textiles of Shandong Province, 308 Ningxia Road, Qingdao 266071, China; 4 State Key Laboratory of Bio-Fibers and Eco-Textiles, Qingdao University, Qingdao 266071, China

**Keywords:** organic solar cells, benzodithiophene, nonfullerene

## Abstract

Using single-bonded and fused aromatic rings are two methods for extending the π-conjugation in the vertical direction of benzo [1,2-b:4,5-b′] dithiophene (BDT) unit. To investigate which method is more efficient in nonfullerene systems, two novel polymers based on alkylthionaphthyl and alkylthiobiphenyl substituted BDT named PBDTNS-FTAZ and PBDTBPS-FTAZ are designed and synthesized. Two polymers only exhibit small differences in structure, but huge differences in photovoltaic properties. They are studied by blended with 3,9-bis(2-methylene-(3-(1,1-dicyanomethylene)indanone)-5,5,11,11-tetrakis(4-hexylphenyl)dithieno [2,3-d’:2,3’-d’]-s-indaceno [1,2-b:5,6-b’] dithiophene (ITIC). The device based on PBDTNS-FTAZ:ITIC showed the best power conversion efficiency (PCE) of 9.63% with the *V*_oc_ of 0.87 V, a *J*_sc_ of 18.06 mA/cm^2^ and a fill factor of 61.21%, while the PBDTBPS-FTAZ:ITIC only exhibit a maximum PCE of 7.79% with a *V*_oc_ of 0.86 V, a *J*_sc_ of 16.24 mA/cm^2^ and a relatively low fill factor of 55.92%. Therefore, extending π-conjugation with alkylthionaphthyl is more effective against constructing nonfullerene solar cells.

## 1. Introduction

Polymer solar cells (PSCs) have attracted tremendous attention from researchers all over the world due to its flexibility, low cost, extensive sources and the possibility for large-scale production [1,2,3,4,5,6]. These features promise a bright commercial prospect. Nonfullerene polymer solar cells have been a research hotspot recently. The power conversion efficiency (PCE) of widely studied n-type semiconductor 3,9-bis(2-methylene-(3-(1,1-dicyanomethylene)-indanone)-5,5,11,11-tetrakis(4-hexylphenyl)-dithieno[2,3-d:2′,3′-d′]-s-indaceno[1,2-b:5,6-b′]-dithiophene (ITIC)-based polymer solar cells have made significant progress in last two years [7,8,9,10,11,12,13,14] because of the advantage of broader absorption in the visible-near infrared region and fine-tuned energy level compared with the traditional PSCs based on fullerene acceptors [15,16,17]. With the development of new polymer donor and modification of nonfullerene acceptor, the PCE of nonfullerene PSCs has reached 13%–18% [3,11,18,19,20,21,22,23,24,25,26,27,28]. Designing and synthesizing novel medium bandgap polymer donors that match well with nonfullerene acceptors are also of vital importance in realizing high-performance solar cells.

There are two strategies to optimize their structure and enhance their photovoltaic performances: backbone engineering and side-chain engineering [3,21]. For backbone engineering, donor-acceptor (D-A) alternating copolymerization has been a widely applied method. Among the variety of donor units, benzo[1,2-*b*:4,5-*b*’]dithiophene (BDT) has been a splendid electron-donating building block due to its favorable planarity and intense π-π interaction [29,30,31,32]. Till now, a large amount of studies has been reported and demonstrated high efficiency. The power conversion efficiency (PCE) of the PSCs with BDT building block has reached 13–18% for single-junction devices [8,18,33,34,35,36,37].

Because the size, distribution and type of side-chains can greatly impact the configuration and properties of the materials, a significant number of trials have been devoted to side-chain engineering to explore high-performance photovoltaic materials [38,39,40]. Hou and coworkers designed two-dimensional BDT (BDTT) by incorporating thiophene conjugated side-chains to the BDT backbone [41], which have superior photovoltaic performance than the alkoxy-substituted BDT (1D-BDT). With the success of BDTT, more 2D-BDT monomers with larger side-chain π-conjugated systems were designed to extend the vertical conjugated length and further enhance π-π stacking. Up to now, conjugated 2D side-chains including thieno [3,2-*b*]thiophene, thiophene, benzene, biphenyl and naphthalene have been studied by many researchers [42,43,44,45,46,47,48]. Besides, the conjugated 2D side-chains can be simply modified by introducing oxygen or sulfur atoms. Interestingly, the sulfur atom possesses some special properties in PSCs. Introducing a sulfur atom could make the absorption of material red-shifted and the HOMO level decline. As a result, polymers containing alkylthio side-chains usually exhibit larger *J*_sc_ and *V*_oc_ [49,50,51,52,53]. As reported, the BDT monomer substituted with alkylthiobiphenyl (BDTBPS) and alkylthionaphthyl (BDTNS) could be promising electron-donating units for designing high-performance donor polymers. Previous reports show the difference between introducing single-bonded and fused aromatic rings into a BDT unit in the fullerene system [48]. Therefore, what are the differences between the single-bonded and fused aromatic rings on aBDT unit in the nonfullerene system? A comparison needs to be conducted.

The fluorinated 2-alkyl-benzo[*d*] [1,2,3] triazoles (FTAZ) and fluorinated benzothiadiazole (ffBT) are two electron-withdrawing units for constructing D-A copolymers with 2D BDT. The TAZ-based polymers showed a higher LUMO energy level than the BT counterparts. Besides, the fluorine atom could pull down both the HOMO and LUMO energy levels of the polymer while holding the bandgap constant, leading to higher *V*_oc_. These make FTAZ more suitable for constructing medium bandgap donor polymer matching with ITIC [53].

Herein, fluorinated 2-alkyl-benzo[d] [1,2,3]triazoles (FTAZ) was chosen as the acceptor building block. The photovoltaic properties of two novel polymers based on BDTNS (named PBDTNS-FTAZ) and BDTBPS (named PBDTBPS-FTAZ) in nonfullerene systems were detailed studied. The PBDTNS-FTAZ:ITIC exhibit higher PCE of 9.64% with a *V*_oc_ of 0.87 V, a *J*_sc_ of 18.06 mA/cm^2^ and a fill factor of 61.21%, while PBDTBPS-FTAZ:ITIC only shows a maximum PCE of 7.78% with a *V*_oc_ of 0.86 V, a *J*_sc_ of 16.24 mA/cm^2^ and a relatively low fill factor of 55.92%.

## 2. Experimental Section

### 2.1. Materials and Reagents

**Scheme 1:**
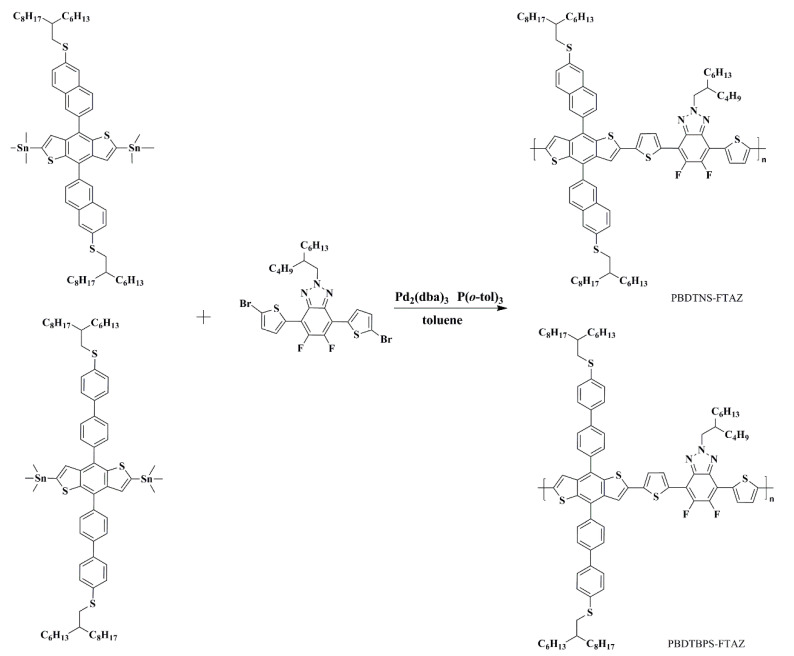
Synthesis route of PBDTNS-FTAZ and PBDTBPS-FTAZ.

All starting materials and reagents were purchased from commercial sources and utilized without further purification. Benzo[1,2-*b*:4,5-*b*’]dithiophene-4,8-dione was purchased from Derthon Optoelectronic Materials Science Technology Co., Ltd., Shenzhen, China, FTAZ were purchased from Solarmer Materials Inc., Irwindale, CA, United States. The purity of all reagents was 99% in the experiment. Toluene was dried over Na/benzophenone and freshly distilled before use. The synthetic routes of polymers PBDTNS-FTAZ and PBDTBPS-FTAZ are shown in Scheme 1.

### 2.2. Synthesis of PBDTNS-FTAZ and PBDTBPS-FTAZ

The compounds BDTNSSn and BDTBPSSn were synthesized as previously reported.

### 2.3. Synthesis of Polymer PBDTNS-FTAZ

Compounds BDTNSSn (128.1 mg, 0.1 mmol), FTAZ-Br (64.5 mg, 0.1 mmol), Pd_2_(dba)_3_ (1.8 mg, 0.002 mmol) and P(*o*-tol)_3_ (3.6 mg, 0.012 mmol) were added into a flask. The flask was subjected to more than three successive cycles of vacuum followed by refilling with argon. Then, 4 mL of toluene was added. The reaction mixture was heated to 110 °C under argon atmosphere. Two hours later, the mixture was cooled to room temperature and polymer PBDTNS-FTAZ was precipitated by the addition of methanol, filtered and purified by Soxhlet extraction with methanol, chloroform and *o*-dichlorobenzene (*o*-DCB), respectively. The *o*-DCB solution was concentrated by evaporation and then precipitated into methanol. The deep purple solid was filtered to yield the desired polymer PBDTPS-FTAZ (118.1 mg, 82% yield). Mn: 46 kDa, PDI: 2.4.

### 2.4. Synthesis of Polymer PBDTBPS-FTAZ

Compounds of BDTBPSSn (133.3 mg, 0.1 mmol), M2 (64.5 mg, 0.1 mmol), Pd_2_(dba)_3_ (1.8 mg, 0.002 mmol) and P(*o*-tol)_3_ (3.6 mg, 0.012 mmol) were added into a flask. The synthetic procedure is the same as the above procedure of PBDTNS-FTAZ. The deep purple PBDTBPS-FTAZ solid was obtained (116.4 mg, 78% yield). Mn: 42 kDa, PDI: 2.2.

### 2.5. Device Characterization

The polymer solar cell was fabricated utilizing the conventional device structure of ITO/PEDOT:PSS/Polymer:ITIC/PFN/Al. The active area of solar cells was 0.1 cm^2^. The ITO coated glasses were cleaned in an ultrasonic machine with ITO detergent, deionized water, acetone and isopropanol sequentially for 20 min each step. The cleaned ITO substrates were treated with oxygen plasma for 6 min and then covered with PEDOT: PSS (Baytron PVP Al 4083) by spin coating. The thickness of PEDOT: PSS was about 35 nm. Then, the substrates were annealed at 160 °C for 20 min. Sequentially, the substrate was transferred to the glove box filled with N_2_ atmosphere. The *o*-DCB solutions of polymers and ITIC were initially heated at 120 °C for 30 min and then stirred at room temperature for 6 h. The solutions were heated at 140 °C half an hour before spin coating. The solutions were hot spin-coated at 140 °C to make the active layer and the substrate was heated at 70 °C. After that, the PFN solutions were spin-coated above the active layer at 2500 rpm for 10 s as the buffer layer. Finally, the samples were transferred to a vacant chamber and Al (100 nm) was deposited in a high vacuum degree (5 × 10^−4^ Pa) via a mask that constrains the active area of 0.1 cm^2^.

The current density–voltage (*J–V*) curves were measured by Keithley 2420 source meter under simulated AM 1.5 G irradiation (100 mW /cm^2^) using a Newport solar simulator. The light intensity was calibrated by a standard silicon solar cell. The external quantum efficiency (EQE) of the solar cells was tested using a certified Newport incident photon conversion efficiency (IPCE) measurement.

Atomic force microscopy (AFM) measurement was performed by an Agilent 5400 with tapping mode. Transmission electron microscopy (TEM) images were obtained from a Hitachi H-7650 transmission electron microscope at an accelerating voltage of 100 kV. The absorption spectra were measured using a Hitachi U-400 UV–vis–NIR scanning spectrophotometer.

## 3. Result and Discussion

### 3.1. Synthesis and Characterization

The synthetic routes of the polymer PBDTNS-FTAZ and PBDTBPS-FTAZ are shown in Scheme 1. The polymers were obtained by a stille coupling reaction. The detailed synthetic procedure is displayed in the Experimental section. The thermal property of the polymers was measured by thermogravimetric analysis (TGA) in nitrogen atmosphere at the heating rate of 10 °C/min. As shown in Figure S1, PBDTNS-FTAZ and PBDTBPS-FTAZ exhibited high thermal stability with an onset decomposition temperature (Td) with a 5% weight loss located at 310 °C.

### 3.2. Optical Properties

**Figure 1:**
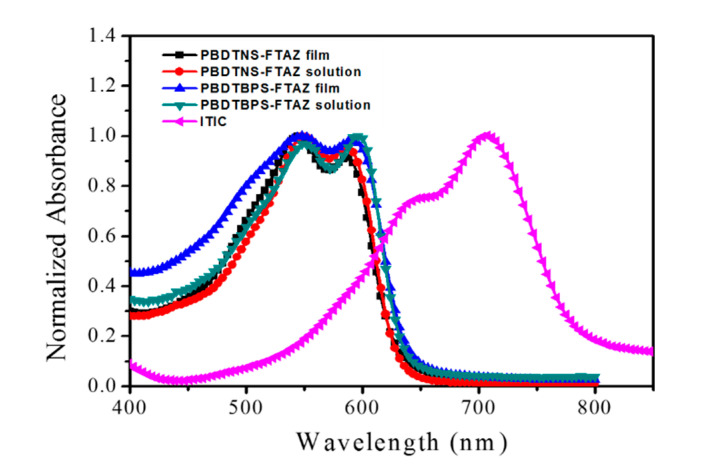
Normalized absorption spectra of PBDTNS-FTAZ, PBDTBPS-FTAZ in *o*-DCB solution and the films of PBDTNS-FTAZ, PBDTBPS-FTAZ and ITIC.

The normalized ultraviolet–visible (UV–vis) absorption spectra of PBDTNS-FTAZ and PBDTBPS-FTAZ in dilute *o*-DCB and as a film are shown in Figure 1. The two polymers show very similar absorption features in both solutions and films. The PBDTNS-FTAZ and PBDTBPS-FTAZ two exhibit shoulder peaks in thin films, which were located at 588 and 597 nm. This could be attributed to the aggregations of the polymer chains. [54] The absorption peak of PBDTNS-FTAZ and PBDTBPS-FTAZ located at 544 and 552 nm, respectively, which can be assigned to the intramolecular charge-transfer between BDT and the FTAZ unit. In the film state, both two polymers exhibit a slight red-shift compared to the absorption spectra in dilute *o*-DCB solution, which could ascribe to the aggregation in the solid-state. The optical bandgaps (E_g_^opt^) of PBDTNS-FTAZ and PBDTBPS-FTAZ are evaluated to be 1.98 and 1.95 according to the equation E_g_^opt^ = 1240/λ, respectively. λ represents the absorption onset of the polymers PBDTNS-FTAZ and PBDTBPS-FTAZ.

### 3.3. Electrochemical Properties

**Figure 2:**
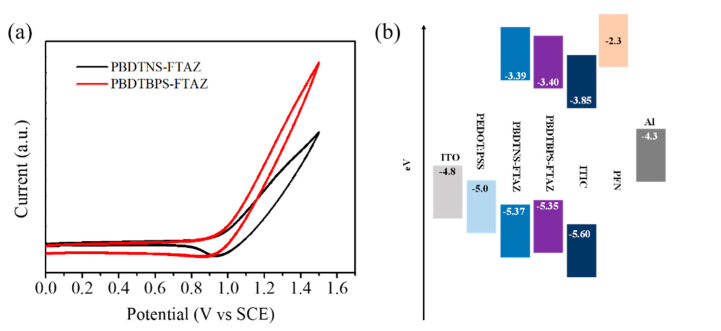
(**a**) Electrochemical cyclic voltammograms of the two polymers, (**b**) the band structure of the device.

The electrochemical properties of PBDTNS-FTAZ and PBDTBPS-FTAZ were characterized by utilizing the cyclic voltammetry (CV) method. The CV curves are shown in Figure 2. The onset oxidation potentials (E_ox_) of PBDTNS-FTAZ and PBDTBPS-FTAZ was 0.97 eV and 0.95 eV vs the saturated calomel electrode (SCE). The calculated HOMO energy levels of PBDTNS-FTAZ and PBDTBPS-FTAZ were calculated to be −5.37 eV and −5.35 eV, respectively. The corresponding LUMO energy levels were −3.39 eV and −3.40 eV calculated by the HOMO energy levels and optical bandgap of the two polymers.

### 3.4. Photovoltaic Properties

**Figure 3:**
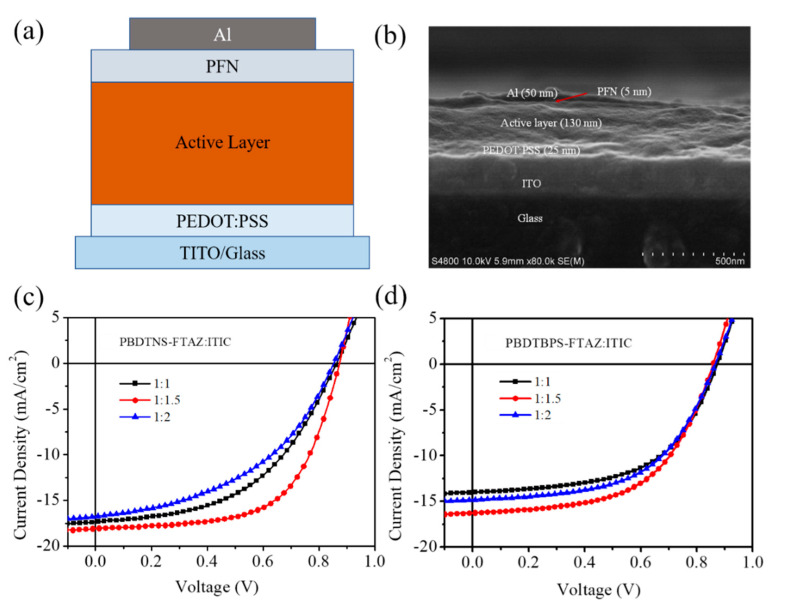
(**a**) Device structure, (**b**) cross-sectional SEM images of the device. *J–V* curves of (**c**) PBDTNS-FTAZ:ITIC and (**d**) PBDTBPS-FTAZ:ITIC PSCs with different D:A ratios.

**Figure 4:**
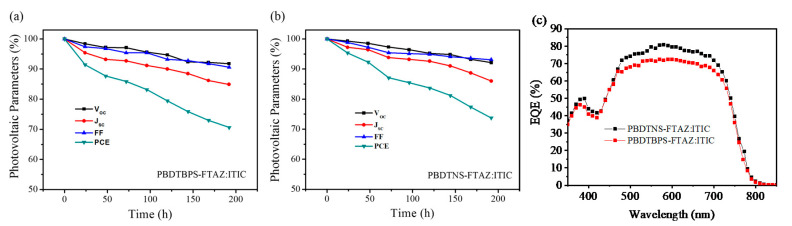
(**a**,**b**) Normalized photovoltaic parameters aged in air for 192 h; (**c**) external quantum efficiency (EQE) spectra of devices based on PBDTNS-FTAZ and PBDTBPS-FTAZ.

To investigate the photovoltaic properties of these two polymers, BHJ polymer solar cells were fabricated using the conventional device structure (ITO/PEDOT:PSS/active layer/PFN/Al). The polymers and ITIC were dissolved in o-DCB. The solutions were initially stirred at 130 °C for one hour and then stirred at room temperature overnight. Devices with different donor/acceptor (D/A) ratios (1:1, 1:1.5, 1:2) were fabricated to explore the best D/A ratio of the blend. Current density–voltage (*J–V*) characteristics of all devices were tested under AM 1.5G illumination. The *J–V* curves are shown in Figure 3 and the corresponding device parameters are summarized in Table 1. From Table 1, it can be concluded that both PBDTNS-FTAZ and PBDTBPS-FTAZ exhibited the best photovoltaic performance when the weight ratio between the polymer and ITIC were 1:1.5. The optimal PCEs (8.17%, 9.64%, 6.49%) of PBDTNS-FTAZ:ITIC were all higher than the PCEs of PBDTBPS-FTAZ:ITIC (7.08%, 7.79%, 7.07%). The PCEs of both polymers did not improve with post thermal annealing or processing additives. The device based on PBDTNS-FTAZ:ITIC showed the best PCE of 9.63% with the *V*_oc_ of 0.87V, a *J*_sc_ of 18.06 mA/cm^2^ and a fill factor of 61.21%, while the PBDTBPS-FTAZ:ITIC only exhibit a maximum PCE of 7.79% with a *V*_oc_ of 0.86V, a *J*_sc_ of 16.24 mA/cm^2^ and a relatively low fill factor of 55.92%. The *V*_oc_, *J*_sc_ and fill factor of PBDTNS-FTAZ:ITIC were promoted simultaneously compared to PBDTBPS-FTAZ:ITIC. In addition, device stability is another important parameter for solar cells. The storage tests were carried out to investigate the ambient stability of PBDTNS-FTAZ and PBDTBPS-FTAZ-based devices (Figure 4a,b). After storage for 192 h in air, the PBDTNS-FTAZ-based devices exhibited more excellent ambient stability than that of PBDTBPS-FTAZ-based devices.

To further investigate the reason higher *J*_sc_ of PBDTNS-FTAZ were obtained compared to PBDTBPS-FTAZ. The external quantum efficiency (EQE) was performed and the curves are displayed in Figure 4c. Both the PBDTNS-FTAZ/ITIC and PBDTBPS-FTAZ/ITIC devices showed broad photoresponse from 470 to 720 nm. The EQE response of the PBDTNS-FTAZ-based device were higher than those of PBDTBPS-FTAZ in the whole spectral range, which could partly account for the higher *J*_sc_ of the PBDTNS-FTAZ-based device. The optimal devices possess a high and flat EQE value of over 65%. The calculated current density of 15.98 mA/cm^2^ and 17.86 mA/cm^2^ were obtained by integrating the EQE spectrum with the standard AM 1.5G solar spectrum, which is consistent with the measured *J*_sc_ value with little error (<5%).

**Figure 5:**
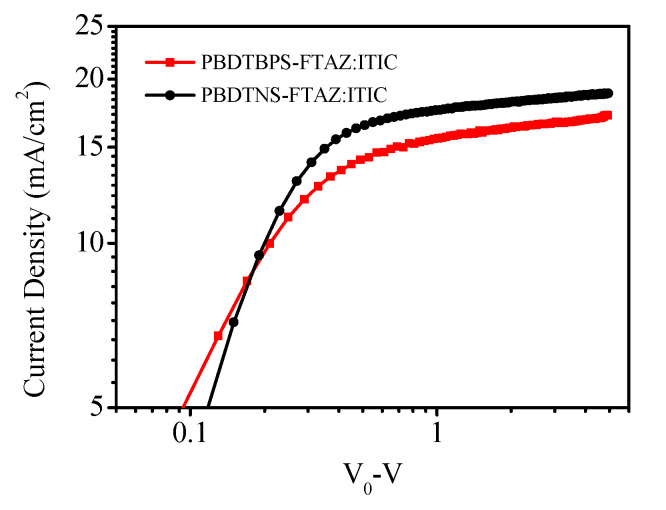
Photocurrent density versus effective voltage (*J*_ph_-*V*_eff_) characteristics.

**Figure 6:**
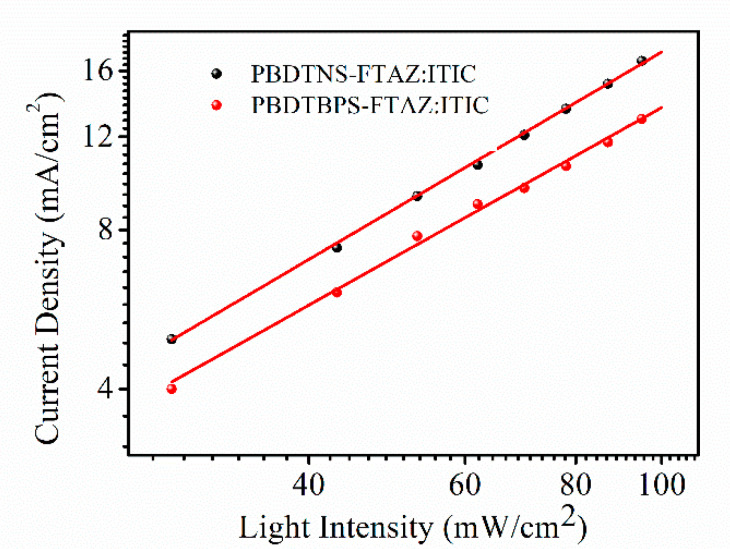
The curve of current density versus incident light intensity.

The relationship of the photocurrent (*J*_ph_) and the effective applied voltage (*V*_eff_) were studied to further analyze the charge recombination process in the devices based on PBDTNS-FTAZ and PBDTBPS-FTAZ. The *J*_ph_ is defined by *J*_L_-*J*_D_, where *J*_L_ and *J*_D_ refer to the current density under AM 1.5G illumination and in the dark, respectively. *V* is the applied voltage; and *V*_0_ refers to the effective voltage where *J*_L_ = *J*_D_ [55,56,57,58,59]. As displayed in Figure 5, the *J*_ph_ of PBDTBPS-FTAZ-based devices could not reach the saturation current (*J*_sat_) even the applied voltage is over 3V, indicating the relatively severe charge recombination in the PBDTBPS-FTAZ:ITIC blend. On the contrary, the *J*_ph_ of the PBDTNS-FTAZ-based devices reached the saturation value (*J*_sat_) at a low applied voltage of 1 V, proving that the photogenerated excitons in PBDTNS-FTAZ-based devices were fully dissociated to free charges. The *J*_sat_ of the PBDTNS-FTAZ-based device was higher than the *J*_sat_ of PBDTBPS-FTAZ-based device, demonstrating that photogenerated current is larger in PBDTNS-FTAZ devices. The charge recombination and exciton dissociation in the PBDTNS-FTAZ:ITIC and PBDTBPS-FTAZ:ITIC device can be further investigated by the *J*_ph_/*J*_sat_ under short-circuit conditions. The *J*_sat_ of PBDTNS-FTAZ and PBDTBPS-FTAZ-based devices were 18.83 mA/cm^2^ and 17.17 mA/cm^2^, respectively. The *J*_ph_/*J*_sat_ of PBDTNS-FTAZ and PBDTBPS-FTAZ-based devices were 97.5% and 94.5%, respectively, proving that the PBDTNS-FTAZ-based device exhibited a better exciton dissociation efficiency at the D/A interfaces and then was collected at the electrodes with little recombination. As shown in Figure 6, the power-law dependence of photocurrent on light intensity (*P*), *J*_sc_ vs. *P*^α^ was plotted. The α value for PBDTNS-FTAZ:ITIC and PBDTBPS-FTAZ:ITIC was 0.985 and 0.938, respectively. The α value approaching one demonstrating there was little bimolecular recombination in PBDTNS-FTAZ:ITIC devices, which could account for the higher *J*_sc_ and FF of the PBDTNS-FTAZ-based devices [60,61,62,63].

### 3.5. Charge Transport Characteristics

The hole mobility of PBDTNS-FTAZ:ITIC and PBDTBPS-FTAZ:ITIC were characterized using the space-charge-limited-current (SCLC) method to study the charge transport ability. The *J–V* curves were shown in Figure S2. The hole only diodes were fabricated with the structure of ITO/PEDOT:PSS/active layer/Au. The hole mobility of PBDTNS-FTAZ and PBDTBPS-FTAZ are 6.64 × 10^−5^ cm^2^/Vs and 2.28 × 10^−5^ cm^2^/Vs, respectively. The PBDTNS-FTAZ exhibited higher hole mobility than PBDTBPS-FTAZ, which may result in a higher *J*_sc_ and FF in the PBDTNS-FTAZ-based devices.

### 3.6. Morphology Characterization

**Figure 7:**
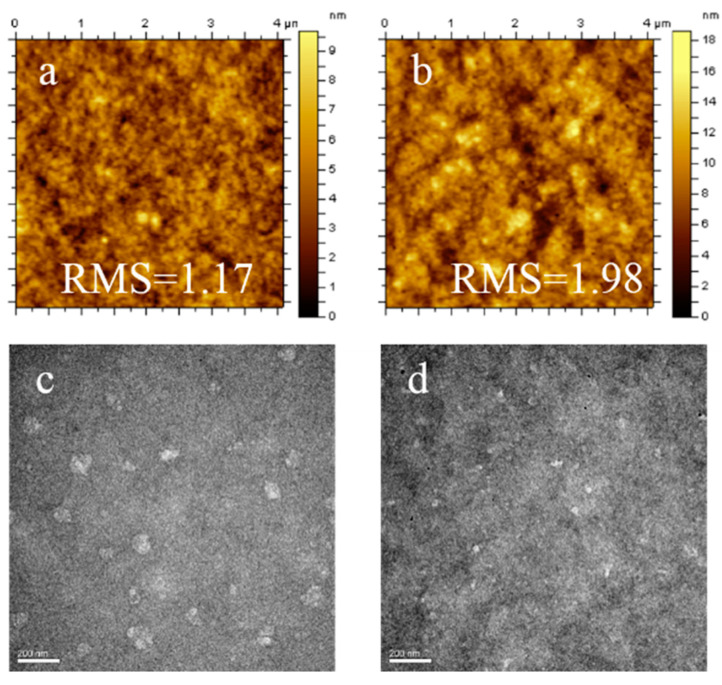
AFM images of (**a**) PBDTNS-FTAZ:ITIC (**b**) PBDTBPS-FTAZ:ITIC and TEM images of (**c**) PBDTNS-FTAZ:ITIC and (**d**) PBDTBPS-FTAZ:ITIC.

Transmission electron microscopy (TEM) and atomic force microscopy (AFM) measurements were performed to look into the surface morphology and the inside bulk structure of the active layer. As shown in Figure 7, the AFM and TEM images could tell that the PBDTNS-FTAZ:ITIC blend film possessed a smooth surface with a relatively low RMS of 1.17 and small aggregated microdomains. Indicating the PBDTNS-FTAZ polymer has excellent miscibility with ITIC. On the contrary, PBDTBPS-FTAZ:ITIC blend film exhibits a rough surface with a large RMS of 1.98 and a large scale of phase separation, indicating the aggregation of polymers and acceptors are severe in the blend film thus limiting the excitons separation and transportation. Thus, we can conclude that the compatibility of PBDTNS-FTAZ:ITIC is much better than that of PBDTBPS-FTAZ:ITIC, resulting in a much better morphology. A favorable morphology is vital for high-performance PSC, which could explain the higher *J*_sc_ and FF obtained in PBDTNS-FTAZ:ITIC device [64].

## 4. Conclusions

In summary, two novel 2D-BDT-based polymers containing alkylthionaphthyl (PBDTNS-FTAZ) and alkylthiobiphenyl (PBDTBPS-FTAZ) as side-chains were synthesized and photovoltaic properties in nonfullerene systems were compared in detail. The PBDTNS-FTAZ exhibited almost the same absorption property as PBDTBPS-FTAZ. However, the PBDTNS-FTAZ shows the better miscibility with ITIC compared with PBDTBPS-FTAZ. Therefore, the PBDTNS-FTAZ exhibited better EQE response, fill factor and yielded a higher PCE of 9.64%. This work proved that extending the side-chain on the BDT unit with fused aromatic rings providing better planarity could facilitate the stacking of polymer, avoiding excessive phase separation and improve PCE when blended with ITIC.

## Figures and Tables

**Table 1:** Device parameters of PBDTNS-FTAZ and PBDTBPS-FTAZ with different polymer:ITIC (D:A) ratios.

*Polymer*	*D/A Ratios (w:w)*	*V_oc_ (V)*	*J_sc_ (mA/cm^2^)*	*FF (%)*	*PCE_max_ (%)*
PBDTNS-FTAZ	1:1	0.88 (0.88 ± 0.01)	15.29 (14.83 ± 0.38)	60.39 (57.12 ± 2.36)	8.17 (7.85 ± 0.24)
	1:1.5	0.87 (0.87 ± 0.01)	18.06 (17.74 ± 0.43)	61.21 (58.19 ± 2.27)	9.64 (9.23 ± 0.36)
	1:2	0.85 (0.85 ± 0.01)	16.70 (16.18 ± 0.54)	45.68 (42.37 ± 3.61)	6.49 (6.12 ± 0.47)
PBDTBPS-FTAZ	1:1	0.87 (0.87 ± 0.01)	14.52 (14.02 ± 0.39)	56.17 (53.89 ± 3.45)	7.08 (6.75 ± 0.31)
	1:1.5	0.86 (0.86 ± 0.01)	16.24 (15.84 ± 0.41)	55.92 (53.86 ± 1.98)	7.79 (7.39 ± 0.56)
	1:2	0.86 (0.86 ± 0.01)	14.82 (14.42 ± 0.58)	55.17 (53.73 ± 1.73)	7.07 (6.46 ± 0.59)

The average values are obtained from 10 devices.

## Supplementary Materials

The following are available online at https://www.mdpi.com/2073-4360/12/8/1673/s1. Figure S1: TGA plots of PBDTNS-FTAZ and PBDTBPS-FTAZ, Figure S2: *J-V* curves of vertical diodes with the device structures of ITO/PEDOT:PSS/polymer: ITIC/Au for hole only devices.
