# Supplementary material for: Comparing Benzodithiophene Unit with Alkylthionaphthyl and Alkylthiobiphenyl Side-Chains in Constructing High-Performance Nonfullerene Solar Cells
Source: Polymers (Basel). 2020 Jul 27;12(8):1673. doi: 10.3390/polym12081673 (PMC7465475; doi:10.3390/polym12081673)
Supplement: Supplementary file 1 [file polymers-12-01673-s001.pdf]

Comparing Benzodithiophene Unit with Alkylthionaphthyl and Alkylthiobiphenyl Side Chains in Constructing High Performance Non-Fullerene Solar Sells

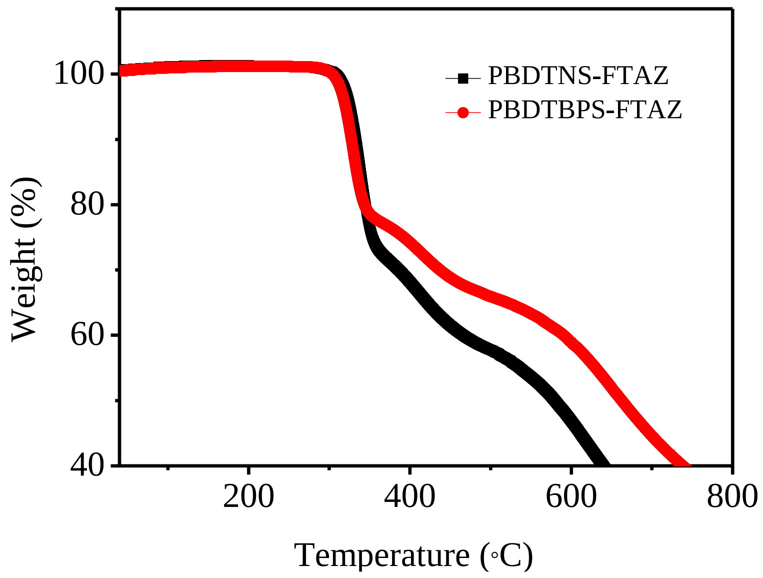

Figure S1. TGA plots of PBDTNS-FTAZ and PBDTBPS-FTAZ.

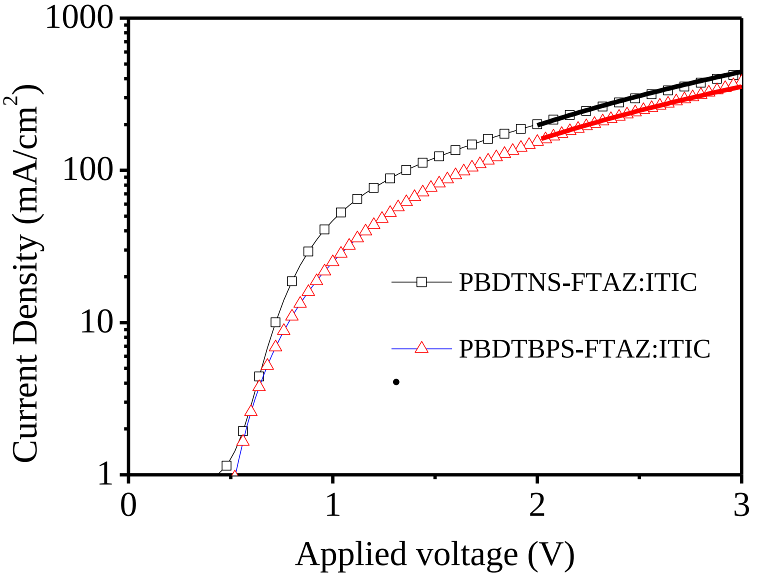

Figure S2. *J-V* curves of vertical diodes with the device structures of ITO/PEDOT:PSS/polymer: ITIC/Au for hole only devices.
